# The association between dietary inflammatory index and cognitive function in adults with/without chronic kidney disease

**DOI:** 10.3389/fnut.2023.1279721

**Published:** 2023-11-21

**Authors:** Biyu Wan, Pinli Lin, Mengya Wang, Jintao Zhong, Lu Peng, Xiaona Tang, Lingzhen Wang, Fang Tang, Yuyan Liang, Xusheng Liu, Lili Deng

**Affiliations:** ^1^School of Nursing Hunan University of Chinese Medicine, Changsha, China; ^2^The Second Clinical College of Guangzhou University of Chinese Medicine, Guangzhou, China; ^3^The Second Affiliated Hospital of Guangzhou University of Chinese Medicine (Guangdong Provincial Hospital of Traditional Chinese Medicine), Guangzhou, China; ^4^Shenzhen Bao'an Traditional Chinese Medicine Hospital Group, Shenzhen, China; ^5^State Key Laboratory of Dampness Syndrome of Chinese Medicine, The Second Affiliated Hospital of Guangzhou University of Chinese Medicine, Guangzhou, China

**Keywords:** aging, dietary inflammatory index, cognitive function, inflammation, diet

## Abstract

**Background and aims:**

Cognitive impairment (CI) is a prevalent condition in patients with chronic kidney disease (CKD), who face an elevated risk of developing cognitive decline. The fundamental mechanism underlying CI is linked to chronic inflammation, which can be gauged by the Dietary Inflammatory Index (DII). The DII is categorized into anti-inflammatory diets with lower scores and pro-inflammatory diets with higher scores. Specifically, pro-inflammatory diets may contribute to chronic inflammation. However, the correlation between the inflammatory potential of diet and cognitive function in patients with CKD has not been explored. This study aims to investigate the connection between the inflammatory potential of diet and cognitive function in individuals with or without chronic kidney disease.

**Methods:**

Data from the 2011–2012 and 2013–2014 National Health and Nutrition Examination Survey (NHANES) were utilized. Participants under the age of 60 or lacking DII, CI, CKD, and other essential data were excluded. DII was computed based on a 24-h dietary recall interview for each participant. Cognitive performance was evaluated using three cognitive tests: the Consortium to Establish a Registry for Alzheimer’s Disease (CERAD) test, the Animal Fluency Test (AFT), and the Digital Symbol Substitution Test (DSST). Logistic regression analysis and subgroup analysis were conducted to assess the independent relationship between DII score and CI in the CKD and non-CKD populations.

**Results:**

The study included a total of 2069 subjects, with CI prevalence ranging from 21.4 to 23.5%. Multiple regression models showed that after adjusting for all covariates of the three cognitive function tests, higher DII scores were significantly associated with increased risk of CI (CERAD OR = 1.18, 95% CI: 1.1 ~ 1.26, AFT OR = 1.15, 95% CI: 1.08 ~ 1.23, DSST OR = 1.19, 95% CI: 1.11 ~ 1.28). Subgroup analysis indicated that the effect of DII score on CI remained consistent in all subgroups (*p* > 0.05).

**Conclusion:**

Higher DII scores were associated with an increased risk of cognitive impairment in people with or without CKD, suggesting that consuming a pro-inflammatory diet may contribute to the impairment of the cognitive function.

## Introduction

CKD is a progressive and irreversible disease characterized by progressive decline of kidney function and damage of kidney structure (1). In 2017, there were nearly 700 million chronic kidney disease patients worldwide, of which 1.2 million died. Furthermore, it is projected that the number of CKD-related deaths will rise to 2 million (and potentially even 4 million) by 2040 (2).

Cognitive function encompasses various mental activities that occur during wakefulness, typically including spatial orientation, learning and memory, numerical aptitude, executive functions, language comprehension, and expression abilities. These abilities generally reflect an individual’ s cognitive state (3). Impairment in any of these cognitive domains is referred to as CI, which represents a state between normal cognition and dementia. Recent reports indicate that over 50 million individuals worldwide are living with dementia, a number expected to surpass 152 million by 2050 (4).

Decline in cognitive function is a frequently overlooked complication of CKD, despite CI and dementia being associated with an elevated risk of mortality (5). Research involving CKD patients has indicated that as kidney function declines, the prevalence and severity of CI increase (6). Conversely, declining cognitive function adversely affects the quality of life and prognosis of kidney disease patients, creating a vicious cycle. Therefore, understanding the mechanism or related factors of CI is of great significance for delaying the progression of CKD. Studies have shown that elevated inflammation is associated with chronic disease and CI, and chronic inflammation has been proposed as a mechanism associated with both. Specifically, higher inflammation levels correspond to elevated levels of interleukin-6 (IL-6) and C-reactive protein (CRP), leading to an accelerated rate of cognitive decline (7). Substantial evidence suggests that various foods, nutrients, and non-nutrient components can influence inflammatory states (8). Dietary inflammatory index (DII) was a literature-derived and population-based scoring system designed to evaluate the inflammatory potential of diets (9). A lower negative DII score suggests an anti-inflammatory effect, while a higher positive DII score means a pro-inflammatory effect of diet (10). Studies have indicated that a pro-inflammatory diet is linked to cognitive decline and contributes to the onset and progression of cognitive impairment and dementia, while adopting an “anti-inflammatory diet” may positively influence cognitive function (11, 12).

In this study, we aimed to investigate the association between DII and cognitive function in patients with or without CKD, utilizing data from the NHANES. Our hypothesis was that an increase in the consumption of pro-inflammatory foods is associated with a decrease in cognitive function levels.

## Materials and methods

### Participants

**Figure 1:**
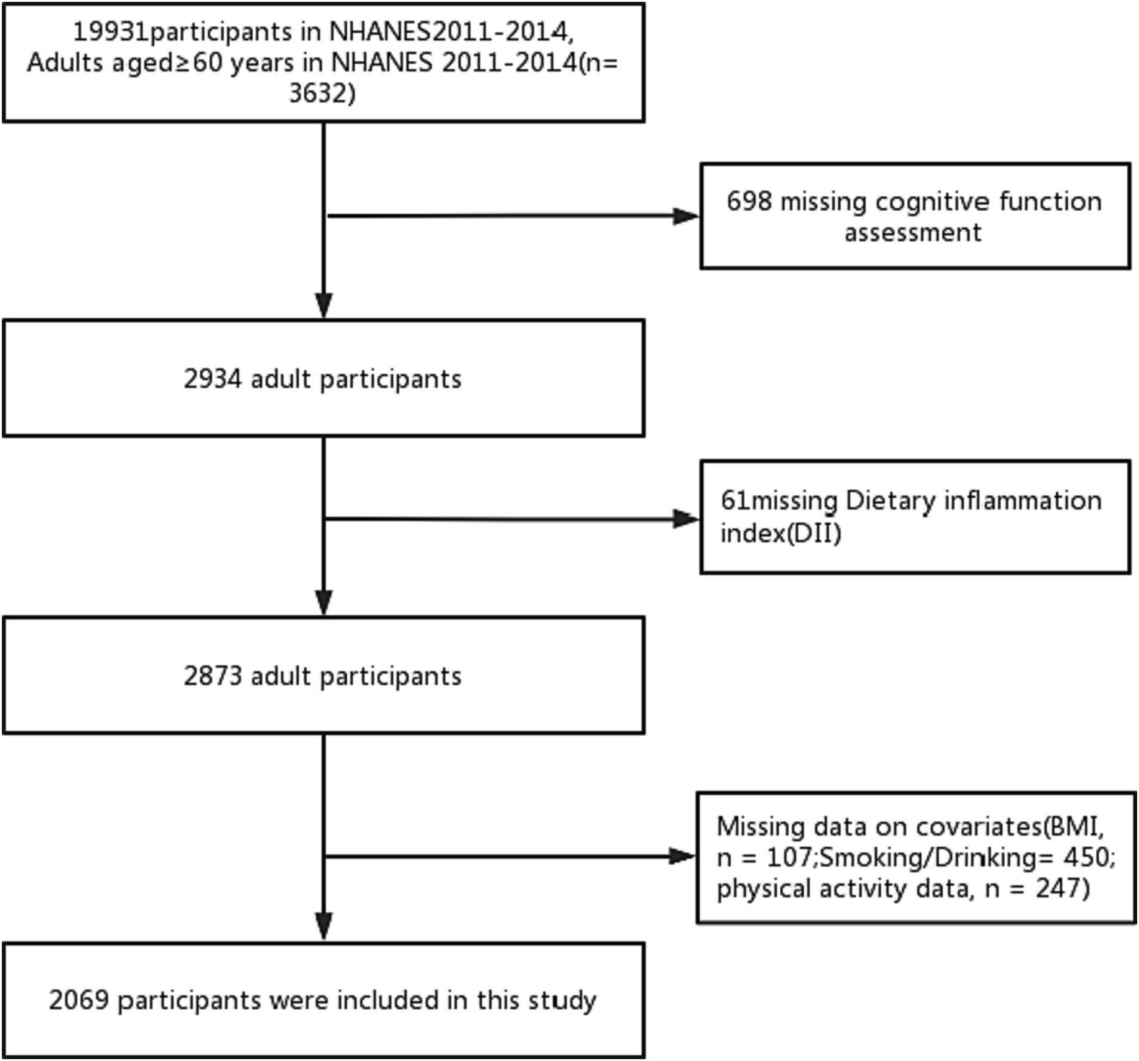
Flowchart of the participants.

Our study is grounded in data from the U.S. Centers for Disease Control’s NHANES database, a continuous project that periodically collects information on the health and nutritional status of the U.S. population. This data is acquired through standardized interviews, physical examinations, and laboratory tests. NHANES is updated every 2 years, and the data is continuously refreshed, ensuring its relevance. In addition, the stratified multi-stage complex probability sampling design is adopted in this study, and the sample size of the study population is highly representative (13). All NHANES data is publicly available at www.cdc.gov/nchs/nhanes/. Our research was conducted based on data obtained from two 2-year NHANES surveys conducted between 2011 and 2014. At the outset, there were a total of 19,931 participants, with 3,632 of them being 60 years or older. In addition, 698 participants did not complete the cognitive function questionnaire, and 61 participants did not finish the 24-h dietary recall interview. After the exclusion of participants with missing data, such as BMI (*n* = 107), smoking and alcohol consumption (*n* = 450), and physical activity (*n* = 247), the final analysis encompassed 2069 participants (Figure 1). The NCHS Ethics Review Committee sanctioned the implementation of NHANES involving human subjects, and all participants provided informed consent.

### Dietary inflammatory index

For a more standardized assessment of the impact of diet on inflammation, DII was developed based on 1,943 articles published from 1950 to 2010, from 11 countries (9). These studies focused on the impacts of 45 food parameters on six inflammatory biomarkers, namely interleukines IL-1β, IL-4, IL-6, IL-10 as well as tumor necrosis factor-alpha (TNF-alpha) and CRP, in order to evaluate an individual’s dietary inflammatory potential (14). It does this by deriving a z-score for each dietary component or nutrient in an individual’s daily intake, relative to the global average and standard deviation of per capita daily intake. The final DII score for an individual is calculated as the sum of the product of these z-scores and the inflammatory effect of the corresponding dietary component or nutrient. Subsequently, the z-score data is standardized, resulting in a symmetric distribution of DII score data with 0 as the midpoint. In this study, 28 different nutrients were employed to compute the DII. These nutrients include alcohol, caffeine, β-carotene, carbohydrate, cholesterol, energy, total fat, fiber, folic acid, iron, magnesium, monounsaturated fatty acids (MUFA), niacin, n-3 fatty acids, n-6 fatty acids, protein, polyunsaturated fatty acids (PUFA), riboflavin, saturated fat, selenium, thiamine, vitamin A, vitamin B12, vitamin B6, vitamin C, vitamin D, vitamin E, and zinc. Meal quality within NHANES was determined based on data from 24-h dietary recall interviews conducted during the interviews. Each NHANES participant is eligible for two 24-h dietary recall interviews. The first interview is conducted in person at the Mobile Examination Center (MEC), and the second interview is performed by telephone 3 to 10 days later. All dietary interviewers undergo an intensive one-week training course and supervised practice interviews prior to independent fieldwork. They are closely monitored during the data collection period. The DII score was analyzed as a continuous variable, and participants were divided into quantiles (low, middle, high) from the total sample for further analysis.

In addition, we controlled for the effect of differences in total energy intake between patients by calculating the DII energy adjusted value (E-DII) consumed per 1,000 kcal/day.

### Cognitive function

Cognitive performance was evaluated using three cognitive tests: CERAD, AFT and DSST.

The CERAD test comprises three consecutive learning trials and one delayed recall test. The final CERAD score is the sum of the three learning trials and the recall test, assessing both immediate and delayed learning. The AFT involves participants naming as many animals as possible in 1 min, scoring one point for each animal named, thus assessing executive function. The DSST is administered on paper and includes a key at the top with 9 pairs of numbers and symbols. Participants are asked to match the corresponding symbols from 133 boxes next to the numbers within 2 min. The total score is based on the number of correct matches, assessing participants’ processing speed, sustained attention, and working memory.

It’s important to note that CERAD, AFT, and DSST do not have universally accepted threshold values for distinguishing CI. However, a prior study (15) using the NHANES database divided the lowest quartile scores in the study population into categories of normal and impaired cognitive function. Consequently, in our study, an overall score below the 25th percentile for each dimension was considered indicative of CI. For this study, the 25th percentile scores for CERAD, AFT, and DSST were 21, 13, and 35, respectively.

### Chronic kidney disease

Urinary albumin-to-creatinine ratio (ACR) was measured during each study period (16). For the purposes of this study, individuals were categorized into two groups: with or without kidney disease. The classification of CKD stages was based on established guidelines: no CKD (ACR < 30 mg/g) and CKD (ACR ≥ 30 mg/g) (17).

### Assessment of covariates

**Figure 2:**
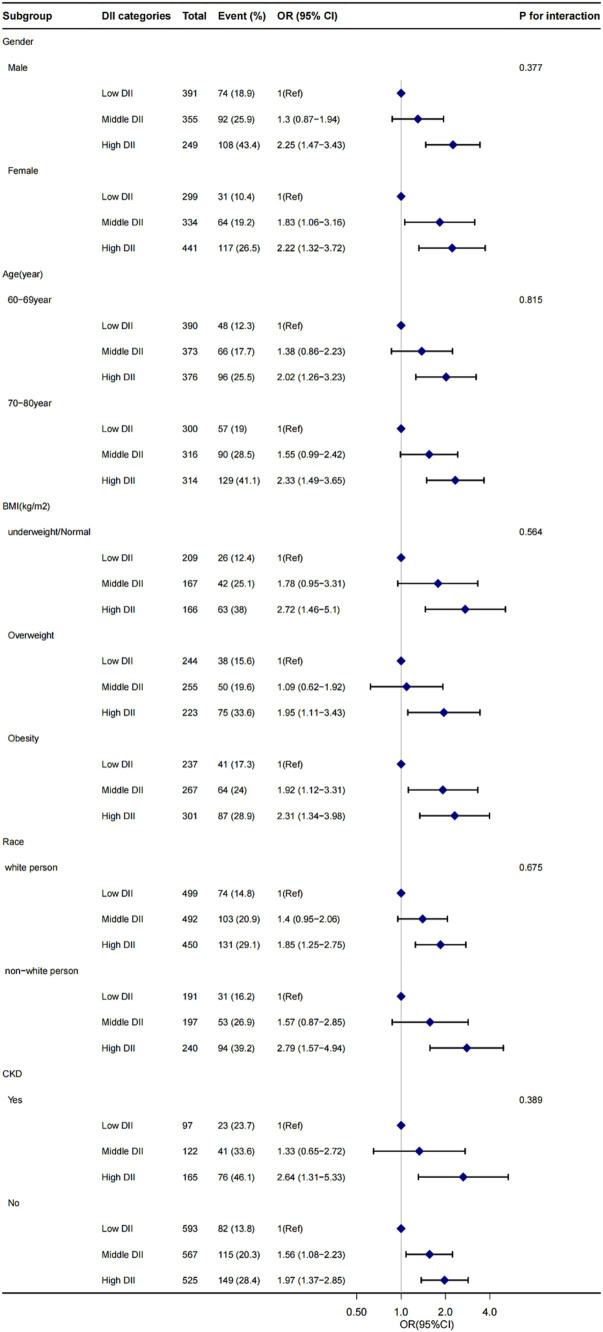
The influence of DII score on CI presence in gender, age, BMI, race, and CKD subgroup. OR, odds ratio; CI, confidence interval. Adjusted for age, gender, race, marital status, drinking status, smoking status, HDL-C, hypertension, diabetes, monthly income, depressive and physical activity. BMI, body mass index; CKD, chronic kidney disease; DII, dietary inflammatory index.

We included several sociodemographic variables in our analysis, including age, race, gender, marital status, education level, and monthly household income. High-density lipoprotein cholesterol (HDL-C) data from blood specimens were analyzed as part of the blood biochemical indices. Monthly household income is reported as specific monthly income ($) and was categorized into three different levels for this study. Body mass index (BMI) was calculated based on height and weight (kg/m^2^). For subgroup analysis, we grouped Mexican-Americans, other Hispanics, and non-Hispanic whites into one category (referred to as “white persons”), and non-Hispanic blacks and other races into another category (referred to as “non-white persons”) (Figure 2). The urinary albumin-to-creatinine ratio (ACR) was calculated, and ACR > 30 mg/g was used to define the presence of renal disease.

Smoking status was classified into three groups: non-smokers (those who smoked <100 cigarettes in their lifetime), ex-smokers (those who had smoked ≥100 cigarettes but currently do not smoke), and current smokers (those who had smoked ≥100 cigarettes and reported the number of cigarettes smoked per day in the past 30 days) (18).

Drinking status was divided into three categories: non-drinkers (those who had consumed <12 drinks of alcoholic beverages in the past year or in their lifetime), former drinkers (those who had consumed ≥12 drinks in their lifetime but not in the past year), and current drinkers (those who had consumed ≥12 drinks in the past year and reported the number of drinks per week).

Hypertension was defined as a mean systolic blood pressure > 140 mmHg or mean diastolic blood pressure > 90 mmHg or self-reported treatment. Diabetes was defined either as a self-report of a physician’s diagnosis of diabetes or screen-detected diabetes.

We assessed depressive symptoms using the Patient Health Questionnaire-9 (PHQ-9). Participants were divided into two groups: those with no depressive symptoms (PHQ-9 score < 10) and those with depressive symptoms (PHQ-9 score ≥ 10).

In addition, participants were categorized into two groups, active (≥ recommended activity level), inactive (< recommended activity level), based on ≥75 min of vigorous or ≥ 150 min of moderate-intensity physical activity per week as recommended by physical activity guidelines (19).

### Statistical analysis

Statistical analyses involved assessing differences between continuous and categorical variables using independent *t*-tests and chi-square tests, respectively. We employed logistic regression models to investigate the association between DII scores and the presence of CI. Multivariate logistic regression models were used to calculate the odds ratios (ORs) and their corresponding 95% confidence intervals (CIs) for the association of elevated DII score with reduced CI. These models included both crude and adjusted versions (Models 1–3). In crude model, no covariates were adjusted. Model 1 was adjusted for gender, age, race and marital status. Model 2 was adjusted for gender, age, race, marital status, body mass index, drinking status, smoking status. Model 3 was adjusted for gender, age, race, marital status, body mass index, drinking status, smoking status, hypertension, diabetes, chronic kidney disease, monthly income, depressive, physical activity and HDL-C.

Subgroup analyses were conducted based on gender, age, BMI, race, and the presence or absence of CKD to explore the relationship between DII score and CI. Additionally, we analyzed the correlation between DII score and CI using box plots.

All statistical analyses were carried out using R 4.1.0, a statistical software package (the R Foundation, Vienna, Austria),^1^ and the Free Statistics platform.

A two-sided *p* < 0.05 was considered statistically significant.

## Results

### Baseline characteristics of participants

The demographic characteristics and other covariates of the included individuals are presented in Table 1. The demographic characteristics and other covariates of the included individuals are presented in Table 1. A total of 2069 subjects were included in the study. They were aged 60 years or older, with 48% being males and 52% females. The participants were categorized into three groups based on DII scores levels: low (−4.90 to 0.15), moderate (0.15–2.05), and high (2.05–4.73). Statistical analysis revealed significant differences between individuals with impaired and normal cognitive performance across all three cognitive function tests. These differences were observed in terms of DII, age, race, monthly household income, alcohol consumption, depression, physical activity, CKD, and HDL-C. However, no statistically significant differences were found in BMI and smoking across the two cognitive function categories.

**Table 1:** Characteristics of the study population, National Health and Nutrition Examination Survey (NHANES) 2011–2014 (*N* = 2,069).

Catalogs	CERAD test			Animal fluency test			Digital symbol substitution test		
	Low cognitive performance	Normal cognitive performance	*p*-value	Low cognitive performance	Normal cognitive performance	*p*-value	Low cognitive performance	Normal cognitive performance	*p*-value
Variables	1 (*n* = 449)	2 (*n* = 1620)		1 (*n* = 443)	2 (*n* = 1626)		1 (*n* = 486)	2 (*n* = 1583)	
Gender (%)			<0.001			0.834			<0.001
Male	274 (61)	721 (44.5)		215 (48.5)	780 (48)		274 (56.4)	721 (45.5)	
Female	175 (39)	899 (55.5)		228 (51.5)	846 (52)		212 (43.6)	862 (54.5)	
Age	72.4 ± 6.9	68.5 ± 6.4	<0.001	71.1 ± 6.9	68.9 ± 6.6	<0.001	71.4 ± 6.9	68.7 ± 6.6	<0.001
Race (%)			0.038			<0.001			<0.001
Mexican American	45 (10)	127 (7.8)		33 (7.4)	139 (8.5)		63 (13)	109 (6.9)	
Other Hispanic	51 (11.4)	143 (8.8)		54 (12.2)	140 (8.6)		90 (18.5)	104 (6.6)	
Non-Hispanic white	224 (49.9)	851 (52.5)		162 (36.6)	913 (56.2)		155 (31.9)	920 (58.1)	
Non-Hispanic black	105 (23.4)	358 (22.1)		153 (34.5)	310 (19.1)		166 (34.2)	297 (18.8)	
Others	24 (5.3)	141 (8.7)		41 (9.3)	124 (7.6)		12 (2.5)	153 (9.7)	
Marital (%)			0.013			0.001			<0.001
Married	236 (52.6)	924 (57)		227 (51.2)	933 (57.4)		230 (47.3)	930 (58.7)	
Widowed	108 (24.1)	277 (17.1)		114 (25.7)	271 (16.7)		130 (26.7)	255 (16.1)	
Divorced	51 (11.4)	244 (15.1)		58 (13.1)	237 (14.6)		61 (12.6)	234 (14.8)	
Separated	15 (3.3)	42 (2.6)		16 (3.6)	41 (2.6)		27 (5.6)	30 (1.9)	
Never married	25 (5.6)	91 (5.6)		21 (4.7)	95 (5.8)		25 (5.1)	91 (5.7)	
Living with partner	14 (3.1)	42 (2.6)		7 (1.6)	49 (3)		13 (2.7)	43 (2.7)	
Hypertension (%)	213 (47.4)	595 (36.7)	<0.001	186 (42)	622 (38.3)	0.153	241 (49.6)	567 (35.8)	<0.001
Diabetes (%)			0.221			0.001			<0.001
Yes	119 (26.5)	356 (22)		129 (29.1)	346 (21.3)		159 (32.7)	316 (20)	
No	310 (69)	1185 (73.1)		295 (66.6)	1200 (73.8)		304 (62.6)	1191 (75.2)	
Boundary value	20 (4.5)	79 (4.9)		19 (4.3)	80 (4.9)		23 (4.7)	76 (4.8)	
Monthly income (%)			<0.001			<0.001			<0.001
0–4,599	233 (51.9)	580 (35.8)		225 (50.8)	588 (36.2)		308 (63.4)	505 (31.9)	
4,600–8,399	130 (29)	526 (32.5)		139 (31.4)	517 (31.8)		123 (25.3)	533 (33.7)	
≧8400	86 (19.2)	514 (31.7)		79 (17.8)	521 (32)		55 (11.3)	545 (34.4)	
BMI	28.5 ± 6.1	29.5 ± 6.5	0.004	28.9 ± 6.3	29.3 ± 6.4	0.259	29.1 ± 6.1	29.3±6.5	0.433
Drinking (%)			0.009			<0.001			<0.001
Never	142 (31.7)	482 (29.8)		153 (34.6)	471 (29)		170 (35.1)	454 (28.7)	
Former	102 (22.8)	283 (17.5)		116 (26.2)	269 (16.5)		133 (27.4)	252 (15.9)	
Current	204 (45.5)	855 (52.8)		173 (39.1)	886 (54.5)		182 (37.5)	877 (55.4)	
Smoking (%)			0.812			0.703			0.011
Never	224 (49.9)	789 (48.8)		216 (48.8)	797 (49.1)		227 (46.8)	786 (49.7)	
Former	174 (38.8)	628 (38.8)		168 (37.9)	634 (39)		180 (37.1)	622 (39.3)	
Current	51 (11.4)	201 (12.4)		59 (13.3)	193 (11.9)		78 (16.1)	174 (11)	
Depressive (%)	55 (12.2)	139 (8.6)	0.018	69 (15.6)	125 (7.7)	<0.001	77 (15.8)	117 (7.4)	<0.001
Physical activity (%)	75 (16.7)	389 (24)	0.001	70 (15.8)	394 (24.2)	<0.001	80 (16.5)	384 (24.3)	<0.001
CKD (%)	338 (75.3)	1347 (83.1)	<0.001	327 (73.8)	1358 (83.5)	<0.001	346 (71.2)	1339 (84.6)	<0.001
HDL-C	52.9 ± 17.0	55.0 ± 16.0	0.015	52.9 ± 16.6	55.0 ± 16.2	0.016	52.7 ± 17.2	55.1±15.9	0.005
DII (%)			<0.001			<0.001			<0.001
Low DII	113 (25.2)	577 (35.6)		101 (22.8)	589 (36.2)		105 (21.6)	585 (37)	
Middle DII	147 (32.7)	542 (33.5)		142 (32.1)	547 (33.6)		156 (32.1)	533 (33.7)	
High DII	189 (42.1)	501 (30.9)		200 (45.1)	490 (30.1)		225 (46.3)	465 (29.4)	

BMI, body mass index; CKD, chronic kidney disease; HDL-C, High-density lipoprotein cholesterol; DII, Dietary Inflammation Index; CERAD, the Consortium to Establish a Registry for Alzheimer’s Disease; *p*-value was tested by Chi-square test or Fisher’s exac.

### Factors associated with CI

Logistic regression analysis was conducted to determine the factors associated with cognitive function within the study population. The results of the regression analysis revealed that several factors, including age, marital status (widowhood), diabetes, kidney disease, monthly family income, HDL-C levels, depression, and physical activity, had significant effects on cognitive function across all three cognitive function tests (*p* < 0.05, as shown in Table 2). Marital status (divorce/unmarried/living with a partner) and smoking status (former smokers) had no specific effects on cognitive function (*p* < 0.05, Table 2).

**Table 2:** Univariate analysis for cognitive function.

Variable	CERAD test		Animal fluency test		Digital symbol substitution test	
	OR (95%CI)	*P*-value	OR (95%CI)	*P*-value	OR (95%CI)	*P*-value
Female	1.95 (1.58–2.42)	<0.001	1.02 (0.83–1.26)	0.834	1.55 (1.26–1.9)	<0.001
Age	0.92 (0.9–0.93)	<0.001	0.95 (0.94–0.97)	<0.001	0.94 (0.93–0.96)	<0.001
Race (%)						
Other Hispanic	0.99 (0.62–1.58)	0.978	0.62 (0.38–1.01)	0.053	0.67 (0.44–1.02)	0.059
Non-Hispanic white	1.35 (0.93–1.95)	0.116	1.34 (0.88–2.03)	0.169	3.43 (2.41–4.89)	<0.001
Non-Hispanic black	1.21 (0.81–1.81)	0.359	0.48 (0.31–0.74)	0.001	1.03 (0.72–1.49)	0.857
Others	2.08 (1.2–3.61)	0.009	0.72 (0.43–1.21)	0.21	7.37 (3.79–14.32)	<0.001
Marriage (%)						
Widowed	0.66 (0.5–0.85)	0.002	0.58 (0.44–0.75)	<0.001	0.49 (0.38–0.63)	<0.001
Divorced	1.22 (0.88–1.71)	0.239	0.99 (0.72–1.37)	0.972	0.95 (0.69–1.3)	0.744
Separated	0.68 (0.37–1.25)	0.218	0.65 (0.35–1.2)	0.165	0.26 (0.15–0.44)	<0.001
Never married	0.93 (0.58–1.48)	0.759	1.1 (0.67–1.8)	0.704	0.9 (0.57–1.43)	0.658
Living with partner	0.77 (0.41–1.43)	0.401	1.7 (0.76–3.81)	0.195	0.82 (0.43–1.55)	0.537
Hypertension (%)	0.64 (0.52–0.79)	<0.001	0.86 (0.69–1.06)	0.154	0.57 (0.46–0.7)	<0.001
Diabetes (%)	1.28 (1–1.63)	0.047	1.52 (1.19–1.93)	0.001	1.97 (1.57–2.48)	<0.001
Boundary value	1.3 (0.76–2.22)	0.33	1.66 (0.96–2.87)	0.072	1.74 (1.04–2.9)	0.034
Monthly income (%)						
4,600–8,399	1.63 (1.27–2.08)	<0.001	1.42 (1.12–1.81)	0.004	2.64 (2.08–3.37)	<0.001
≧8400	2.4 (1.82–3.16)	<0.001	2.52 (1.9–3.35)	<0.001	6.04 (4.43–8.25)	<0.001
CKD (%)	1.62 (1.26–2.08)	<0.001	1.8 (1.4–2.31)	<0.001	2.22 (1.75–2.82)	<0.001
HDL-C	1.01 (1–1.02)	0.015	1.01 (1–1.02)	0.016	1.01 (1–1.02)	0.005
BMI	1.03 (1.01–1.04)	0.004	1.01 (0.99–1.03)	0.259	1.01 (0.99–1.02)	0.433
Drinking (%)						
Former	0.82 (0.61–1.1)	0.178	0.75 (0.57–1)	0.051	0.71 (0.54–0.93)	0.014
Current	1.23 (0.97–1.57)	0.087	1.66 (1.3–2.12)	<0.001	1.8 (1.42–2.29)	<0.001
Smoking (%)						
Former	1.02 (0.82–1.28)	0.831	1.02 (0.82–1.28)	0.846	1 (0.8–1.25)	0.986
Current	1.12 (0.8–1.57)	0.519	0.89 (0.64–1.23)	0.472	0.64 (0.47–0.87)	0.005
Depressive (%)	0.67 (0.48–0.94)	0.019	0.45 (0.33–0.62)	<0.001	0.42 (0.31–0.58)	<0.001
Physical activity (%)	1.58 (1.2–2.07)	0.001	1.7 (1.29–2.25)	<0.001	1.63 (1.25–2.12)	<0.001

CKD, chronic kidney disease; HDL-C, High-density lipoprotein cholesterol; BMI, body mass index; CERAD, the Consortium to Establish a Registry for Alzheimer’s Disease.

### Multivariate logistic regression analysis of the influence of DII scores on cognitive function

The correlation between DII score and cognitive function in the three different cognitive tests was assessed through multivariate regression analysis (Table 3). The analysis indicated that increasing levels of DII scores were associated with an elevated risk of CI across all three tests (CERAD, AFT, and DSST), and these findings were statistically significant (CERAD: Model 1, OR = 1.19, 95% CI: 1.12, 1.27, *p* < 0.001; Model 2, OR = 1.19, 95% CI: 1.12, 1.27, *p* < 0.001; Model 3, OR = 1.18, 95%CI: 1.1, 1.26, *p* < 0.001), (Animal fluency test: Model 1, OR = 1.19, 95%CI: 1.12, 1.27, *p* < 0.001; Model 2, OR = 1.18, 95%CI: 1.11, 1.26, *p* < 0.001; Model 3,OR = 1.15, 95% CI: 1.08, 1.23, *p* < 0.001), (DSST: Model 1, OR = 1.25, 95% CI: 1.17, 1.33, *p* < 0.001; Model 2, OR = 1.21, 95% CI: 1.14, 1.3, *p* < 0.001; Model 3, OR = 1.19, 95% CI: 1.11, 1.28, *p* < 0.001). In Model 3, which was adjusted for all covariates, the results revealed that each unit increase in the DII score was associated with an 18, 19, and 15% increased risk of CI in the three tests, respectively. This association remained statistically significant even after grouping the DII score. Specifically, the fully adjusted effect sizes for the Middle DII score group (Reference: low DII score) were 1.34 (95% CI: 1, 1.79, *p* = 0.051), 1.37 (95% CI: 1.02, 1.85, *p* = 0.038), and 1.46 (95% CI: 1.06, 2.01, *p* = 0.02). Similarly, the fully adjusted effect sizes for the High DII score group were 1.99 (95% CI: 1.48, 2.67, *p* < 0.001), 1.82 (95% CI: 1.3, 2.44, *p* < 0.001), and 2.14 (95% CI: 1.56, 2.94, *p* < 0.001), respectively. In summary, the risk of CI was observed to increase with higher DII scores levels in all crude and adjusted models (Models 1–3). In addition, when analyzed using the E-DII scores, we obtained results similar to those obtained when using the DII scores analysis (Supplementary Tables S1, S2). The value of Akaike information criterion (AIC) tends to decrease with the gradual adjustment of models (Models 1–3), as shown in Table 3.

**Table 3:** Relationship between DII score and cognitive function.

Variable	Crude model			Model 1			Model 2			Model 3		
	OR (95% CI)	AIC	*P*-value	OR (95% CI)	*P*-value	AIC	OR (95% CI)	*P*-value	AIC	OR (95% CI)	*P*-value	AIC
CERAD test												
DII (continuous)	1.16 (1.09–1.23)	2142.43	<0.001	1.19 (1.12–1.27)	<0.001	1965.36	1.19 (1.12–1.27)	<0.001	1958.84	1.18 (1.1–1.26)	<0.001	1936.66
DII categories		2143.82				1970.19			1964.29			1942.05
Low DII	Ref.			Ref.			Ref.			Ref.		
Middle DII	1.38 (1.06–1.82)		0.019	1.38 (1.03–1.83)	0.029		1.37 (1.03–1.83)	0.031		1.34 (1–1.79)	0.051	
High DII	1.93 (1.48–2.5)		<0.001	2.1 (1.58–2.79)	<0.001		2.11 (1.58–2.81)	<0.001		1.99 (1.48–2.67)	<0.001	
Trend test			<0.001		<0.001			<0.001			<0.001	
Animal fluency test												
DII (continuous)	1.22 (1.15–1.3)	2105.86	<0.001	1.19 (1.12–1.27)	<0.001	1987.74	1.18 (1.11–1.26)	<0.001	1979.36	1.15 (1.08–1.23)	<0.001	1943.57
DII categories		2110.45				1993.7			1984.9			1948.65
Low DII	Ref.			Ref.			Ref.			Ref.		
Middle DII	1.51 (1.14–2)		0.004	1.44 (1.08–1.93)	0.013		1.42 (1.06–1.91)	0.018		1.37 (1.02–1.85)	0.038	
High DII	2.38 (1.82–3.11)		<0.001	2.11 (1.59–2.8)	<0.001		2.02 (1.51–2.69)	<0.001		1.82 (1.35–2.44)	<0.001	
Trend test			<0.001		<0.001			<0.001			<0.001	
Digital symbol substitution test												
DII (continuous)	1.25 (1.18–1.32)	2198.19	<0.001	1.25 (1.17–1.33)	<0.001	1850.29	1.21 (1.14–1.3)	<0.001	1827.56	1.19 (1.11–1.28)	<0.001	1721.12
DII categories		2200.92				1854.1			1830.05			1724.23
Low DII	Ref.			Ref.			Ref.			Ref.		
Middle DII	1.63 (1.24–2.14)		<0.001	1.58 (1.17–2.14)	0.003		1.53 (1.13–2.08)	0.006		1.46 (1.06–2.01)	0.02	
High DII	2.7 (2.08–3.5)		<0.001	2.64 (1.97–3.55)	<0.001		2.39 (1.77–3.23)	<0.001		2.14 (1.56–2.94)	<0.001	
Trend test			<0.001		<0.001			<0.001			<0.001	

Crude Model: without covariates adjustment. Model 1 = Age + Gender + Race + Marital status. Model 2 = Model1 + BMI + Drinking status + Smoking status. Model 3 = Model1 + Model2 + CKD + HDL-C + Hypertension + Diabetes + Monthly income + Depressive + Physical activity. The bold values indicate statistically significant values of *p* < 0.05. DII, dietary inflammatory index; CERAD, Consortium to Establish a Registry for Alzheimer’s disease; AIC, Akaike information criterion.

**Figure 3:**
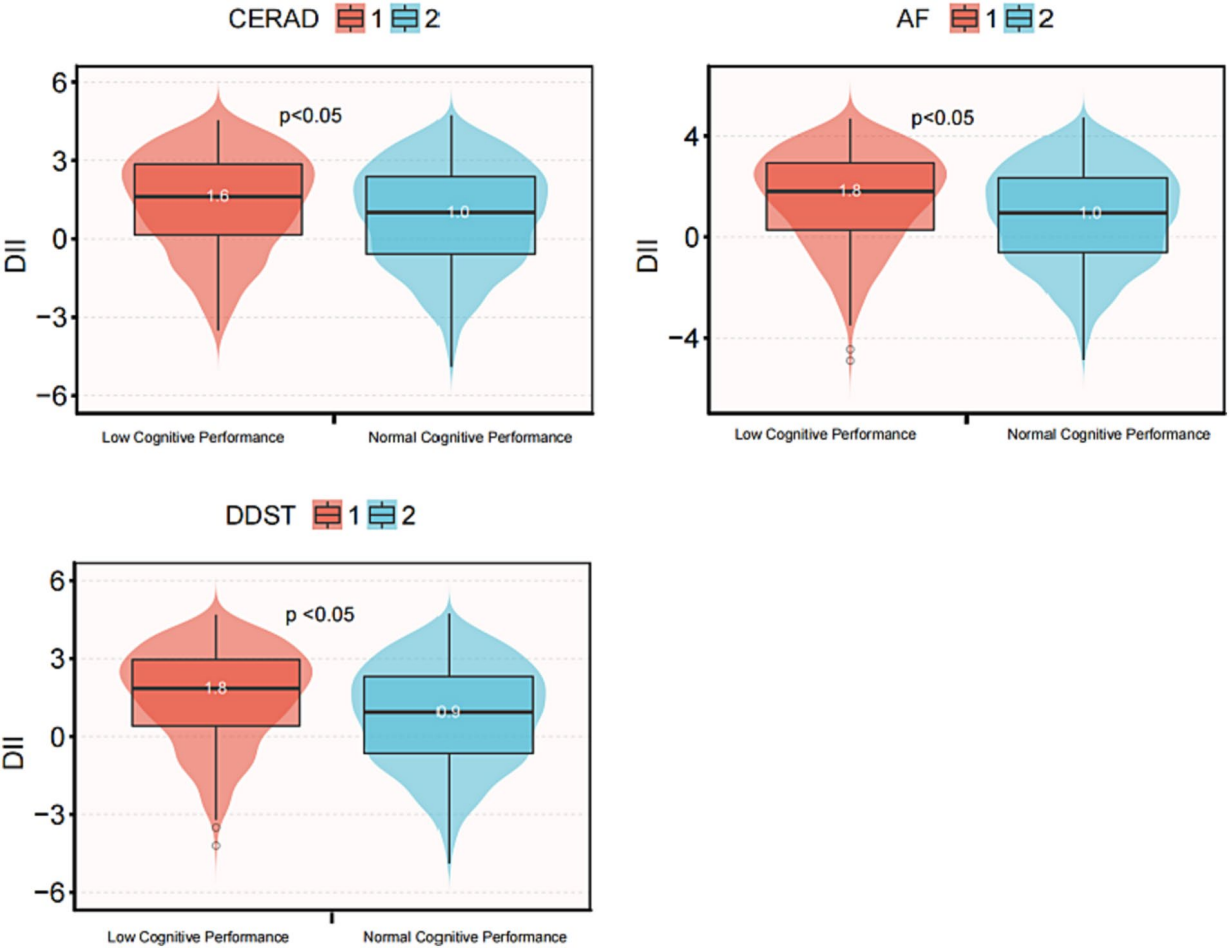
Box diagram of DII score and cognitive function in three cognitive function tests. DII, Dietary Inflammatory Index; CERAD, Consortium to Establish a Registry for Alzheimer’s disease; AFT, Animal Fluency test; DSST, Digital Symbol Substitution Test. The scores of CERAD, AFT and DSST below 21, 13, and 35, respectively, are considered low cognitive performance, otherwise they are considered normal cognitive performance.

To further validate the association between DII score and cognitive function, a box plot analysis was employed, as illustrated in Figure 3. Number 1 and depict low and normal cognitive function, respectively. In all three cognitive function tests, the median DII score for low cognitive function was significantly higher than that for normal cognitive function, and this difference was statistically significant (*p* < 0.05).

### Subgroup analyses

Stratified analyses were conducted to assess the stability of the relationship between DII scores and cognitive function across various subgroups. These subgroups were defined by gender (female and male), age (60–69 and 70–80 years old), BMI (<25 kg/m^2^, 25 ≤ BMI < 30 kg/m^2^, and ≥ 30 kg/m^2^), race (white person, non-white person), and CKD (with or without). The results indicated that none of these variables significantly affected the association between DII scores and cognitive functioning (all *p* > 0.05 for interaction), as shown in Figure 2.

## Discussion

In this cross-sectional study involving 2069 adults, we observed a significant positive association between higher DII scores and CI, this suggests that a highly pro-inflammatory diet may increase the risk of CI. Even after adjusting for various covariates, including age, gender, race, marital status, drinking status, smoking status, HDL-C, hypertension, diabetes, monthly income, depressive symptoms, and physical activity, the relationship between higher DII scores and CI remained robust. Subgroup analysis stratified by the renal condition and other variables showed that this positive association was not affected, suggesting that this association could be appropriate for the population with different renal function conditions, gender, age, BMI, and race (Figure 2).

The CI is a common occurrence in CKD, and although the exact mechanisms are not yet fully understood, factors such as oxidative stress and chronic inflammation are believed to play a pivotal role (20). The DII is a tool that links diet with inflammation. A recent meta-analysis suggests a connection between a higher DII score, indicating a more pro-inflammatory diet, and an elevated risk of CI (21), which aligns with the findings of this study. Additionally, there is a growing body of evidence indicating that inflammation is strongly associated with CI and the risk of developing dementia. Chronic and excessive inflammatory responses may contribute to the progression of CI (22). Consuming a pro-inflammatory diet is known to be a key factor in the presence of these inflammatory markers, with dietary choices significantly impacting chronic systemic inflammation levels (23). Different nutrients in food have inconsistent effects on blood markers of inflammation, with some having pro-inflammatory and others anti-inflammatory effects (24). For example, dietary polyphenol intake reduces the levels of serum inflammatory markers such as vascular cell adhesion molecule-1, intercellular adhesion molecule-1, IL-6, TNF-alpha, and monocyte chemotactic protein-1; and DHA intake reduces the levels of IL-1. The intake of saturated fatty acids, trans fatty acids, etc. increases serum levels of inflammatory markers. It has been shown that inflammatory variables in plasma are affected by different food intakes. For example, with a decrease in IL-1β/IL-6 (cardiac inflammatory factors) and an increase in IL-6 and IL-8 when beef is consumed compared to the habitual diet. Oxygen free radicals, which drive somatic inflammatory factors, are usually associated with increased inflammation, suggesting that pro-inflammatory diets lead to plasma inflammation (25). In addition, a study of Korean older adults mentioned (26) that consumption of a pro-inflammatory diet was associated with an increased risk of CI. However, despite the literature suggesting the effect of pro-inflammatory diets on CI, there are contrary findings that suggest no significant association between DII scores and CI (18, 27). Inconsistency may be due to differences in study populations, methodology, or dietary scoring methods (28, 29). The aim of this study was to explore the role of DII score on the risk of CI by analyzing the NHANES database in patients with/without chronic kidney disease.

To the best of our knowledge, this is the first comprehensive study to investigate the relationship between DII score and cognitive function in older Americans (Table 2). The results across three different cognitive function tests consistently demonstrated a progressive increase in the risk of CI with higher DII scores. In the fully adjusted model, the risk of developing CI was notably higher with high DII score compared to low DII score (Table 2). Furthermore, the relationship between DII score and cognitive function remained stable across different subgroups, as indicated by the stratified analysis (Figure 2).

The DII is a quantitative measure of pro-inflammatory diet, which assesses an individual’s dietary inflammatory potential based on the effects of 45 food parameters on six inflammatory biomarkers (IL-1β, IL-4, IL-6, IL-10, TNF-alpha, and CRP) (30) and correlates with systemic markers of inflammation, including tumor necrosis factor (TNF), interleukin (IL), and interferon (IFN-γ) (31). Persistently elevated levels of inflammation are associated with neurodegeneration and chronic disease (32). Studies have demonstrated that overexpression of TNF-alpha can trigger chronic central nervous system (CNS) inflammation and white matter degeneration (33). Pathologic accumulation of Aβ is a key factor driving neuroinflammatory responses in patients with Alzheimer’s disease (34). *In vitro*, IL-1β can promote Aβ deposition by regulating the expression and protein hydrolysis of the amyloid precursor protein (APP) front. If prolonged inflammation will damage the blood–brain barrier, these inflammatory cells (IL-1β, IL-6, and TNF-alpha) can cross the blood–brain barrier and predispose to brain impairment thus affecting cognitive function (35, 36). Meanwhile, microglia, as a major regulator of the inflammatory response in the central nervous system, are essential for maintaining brain homeostasis, and their activation is characterized and regulated by cytokines such as TNF-alpha (37, 38). Studies have shown that higher DII scores, indicative of a more inflammatory diet, are associated with elevated levels of inflammation markers such as IL-6, CRP, and TNF-alpha, all of which are linked to CI (26, 39). Thus, controlling inflammatory factors through dietary choices may play a significant role in delaying cognitive decline.

This study boasts several strengths. Firstly, our sample of older adults was drawn from the nationally representative NHANES database, renowned for its high-quality survey methodology and stringent quality control. Secondly, the inclusion of three different cognitive functioning test scores provided a comprehensive understanding of the relationship between DII scores and CI. However, there are limitations to this study, including its cross-sectional nature, which cannot establish causality and calls for clinical trials (preferably multi-center) to confirm these findings. Additionally, self-reported dietary recall might introduce recall bias, since a single 24-h dietary recall only captures dietary intake for 1 day, and an individual’s diet may vary significantly from day to day. To minimize the effect of one-time recall bias and to ensure the accuracy of dietary information, we performed the analysis using the average of two 24-h dietary recalls for participants. Lastly, DII scores calculation relies on 45 food parameters, but only 28 common parameters were included in this study, warranting further large-scale prospective investigations for validation.

## Conclusion

In this cross-sectional study comprising 2,069 adults, a noteworthy positive association between DII score and cognitive function was discerned. This observation implies that a higher DII scores, indicative of greater pro-inflammatory dietary potential, is linked with an augmented risk of CI. Importantly, this association was consistent among both CKD and non-CKD populations. More data from clinical trials are needed to verify the association.

## Data availability statement

Publicly available datasets were analyzed in this study. This data can be found at: www.cdc.gov/nchs/nhanes/.

## Author contributions

BW: Conceptualization, Data curation, Formal analysis, Writing – original draft, Writing – review & editing. PL: Data curation, Formal analysis, Writing – review & editing. MW: Data curation, Formal analysis, Methodology, Writing – review & editing. JZ: Data curation, Formal analysis, Writing – review & editing. LP: Conceptualization, Writing – review & editing. XT: Funding acquisition, Writing – review & editing.

LW: Formal analysis, Writing – review & editing. FT: Formal analysis, Methodology, Writing – review & editing. YL: Writing – review & editing. XL: Funding acquisition, Formal analysis, Writing – review & editing. LD: Formal analysis, Supervision, Writing – review & editing.

## Footnotes

^1^ http://www.R-project.org
