# Supplementary material for: The association between dietary inflammatory index and cognitive function in adults with/without chronic kidney disease
Source: Front Nutr. 2023 Nov 21;10:1279721. doi: 10.3389/fnut.2023.1279721 (PMC10703050; doi:10.3389/fnut.2023.1279721)

Supplementary table 1. Relationship between E-DII score and Cognitive Function.

CERAD test

Variable	Crude model		Model1		Model2		Model3	
	OR(95% CI)	P-value	OR(95% CI)	P-value	OR(95% CI)	P-value	OR(95% CI)	P-value
E-DII(continuous)	1.15 (1.08~1.22)	<0.001	1.18 (1.11~1.26)	<0.001	1.19 (1.11~1.27)	<0.001	1.17 (1.1~1.26)	<0.001
E-DII categories								
Low E-DII	Ref.		Ref.		Ref.		Ref.	
Middle E-DII	1.23 (0.93~1.61)	0.142	1.21 (0.91~1.62)	0.184	1.22 (0.91~1.63)	0.176	1.18 (0.88~1.58)	0.266
High E-DII	1.92 (1.48~2.48)	<0.001	2.14 (1.61~2.85)	<0.001	2.15 (1.61~2.88)	<0.001	2.07 (1.54~2.79)	<0.001
Trend test		<0.001		<0.001		<0.001		<0.001

Animal fluency test

Variable	Crude model		Model1		Model2		Model3	
	OR(95% CI)	P-value	OR(95% CI)	P-value	OR(95% CI)	P-value	OR(95% CI)	P-value
E-DII(continuous)	1.23 (1.16~1.3)	<0.001	1.2 (1.13~1.28)	<0.001	1.19 (1.12~1.27)	<0.001	1.17 (1.09~1.25)	<0.001
E-DII categories								
Low E-DII	Ref.		Ref.		Ref.		Ref.	
Middle E-DII	1.47 (1.11~1.95)	0.007	1.41 (1.05~1.89)	0.021	1.39 (1.04~1.87)	0.027	1.35 (1~1.81)	0.051
High E-DII	2.43 (1.86~3.18)	<0.001	2.11 (1.59~2.8)	<0.001	2.01 (1.51~2.69)	<0.001	1.84 (1.37~2.48)	<0.001
Trend test		<0.001		<0.001		<0.001		<0.001

Digital symbol substitution test

Variable	Crude model		Model1		Model2		Model3	
	OR(95% CI)	P-value	OR(95% CI)	P-value	OR(95% CI)	P-value	OR(95% CI)	P-value
E-DII(continuous)	1.28 (1.2~1.35)	<0.001	1.29 (1.2~1.38)	<0.001	1.25 (1.17~1.34)	<0.001	1.22 (1.14~1.32)	<0.001

E-DII categories								
Low E-DII	Ref.		Ref.		Ref.		Ref.	
Middle E-DII	1.63 (1.24~2.14)	<0.001	1.56 (1.16~2.11)	0.004	1.53 (1.13~2.07)	0.006	1.46 (1.07~2.01)	0.018
High E-DII	2.7 (2.08~3.5)	<0.001	2.68 (1.99~3.6)	<0.001	2.41 (1.78~3.27)	<0.001	2.24 (1.62~3.08)	<0.001
Trend test		<0.001		<0.001		<0.001		<0.001

Crude Model: without covariates adjustment.

Model 1 = Age+ Gender+ Race+ Marital status

Model 2=Model1+BMI+ Drinking status+ Smoking status

Model 3=Model1+ Model2+ CKD+ HDL-C+ Hypertension+ Diabetes+ Income Month+ Depressive+ Physical activity

The bold values indicate statistically significant values of p< 0.05.

Abbreviations: E-DII, energy-adjusted Dietary Inflammatory Index; CERAD, Consortium to Establish a Registry for Alzheimer' s disease.

Supplementary table 2. The influence of E-DII score on CI presence in gender, age, BMI, race, and CKD subgroup. OR, odds ratio; CI, confidence interval. Adjusted for age, gender, race, marital status, drinking status, smoking status, HDL-C, hypertension, diabetes, monthly income, depressive and physical activity. BMI, body mass index. CKD, chronic kidney disease. E-DII, energy-adjusted Dietary Inflammatory Index.

Subgroup	E−DII categories	Total	Event (%)	OR (95%CI)	P for interaction
Gender					
Male					0.886
	Low E−DII	402	73 (18.2)	1(Ref)	
	Middle E−DII	365	104 (28.5)	1.55 (1.04−2.29)	
	High E−DII	228	97 (42.5)	2.24 (1.45−3.46)	
Female					
	Low E−DII	288	32 (11.1)	1(Ref)	
	Middle E−DII	324	52 (16)	1.35 (0.78−2.35)	
	High E−DII	462	128 (27.7)	2.23 (1.34−3.7)	
Age(year)					
60−69year					0.609
	Low E−DII	391	49 (12.5)	1(Ref)	
	Middle E−DII	387	68 (17.6)	1.28 (0.8−2.04)	
	High E−DII	361	93 (25.8)	2.2 (1.36−3.55)	
70−80year					
	Low E−DII	299	56 (18.7)	1(Ref)	
	Middle E−DII	302	88 (29.1)	1.67 (1.07−2.63)	
	High E−DII	329	132 (40.1)	2.36 (1.51−3.68)	
BMI(kg/m2)					
underweight/Normal					0.737
	Low E−DII	205	25 (12.2)	1(Ref)	
	Middle E−DII	165	42 (25.5)	1.73 (0.93−3.23)	
	High E−DII	172	64 (37.2)	3.1 (1.64−5.84)	
Overweight					
	Low E−DII	247	38 (15.4)	1(Ref)	
	Middle E−DII	256	55 (21.5)	1.22 (0.7−2.13)	
	High E−DII	219	70 (32)	1.98 (1.12−3.5)	
Obesity					
	Low E−DII	238	42 (17.6)	1(Ref)	
	Middle E−DII	268	59 (22)	1.68 (0.98−2.89)	
	High E−DII	299	91 (30.4)	2.34 (1.36−4.02)	
Race					
Hispanic					0.292
	Low E−DII	501	75 (15)	1(Ref)	
	Middle E−DII	493	105 (21.3)	1.41 (0.96−2.06)	
	High E−DII	447	128 (28.6)	1.83 (1.23−2.72)	
Non−Hispanic					
	Low E−DII	189	30 (15.9)	1(Ref)	
	Middle E−DII	196	51 (26)	1.58 (0.87−2.87)	
	High E−DII	243	97 (39.9)	3.35 (1.88−5.97)	
CKD					
Yes					0.71
	Low E−DII	101	25 (24.8)	1(Ref)	
	Middle E−DII	118	39 (33.1)	1.42 (0.7−2.87)	
	High E−DII	165	76 (46.1)	2.46 (1.24−4.89)	
No					
	Low E−DII	589	80 (13.6)	1(Ref)	
	Middle E−DII	571	117 (20.5)	1.54 (1.07−2.21)	
	High E−DII	525	149 (28.4)	2.14 (1.47−3.1)	

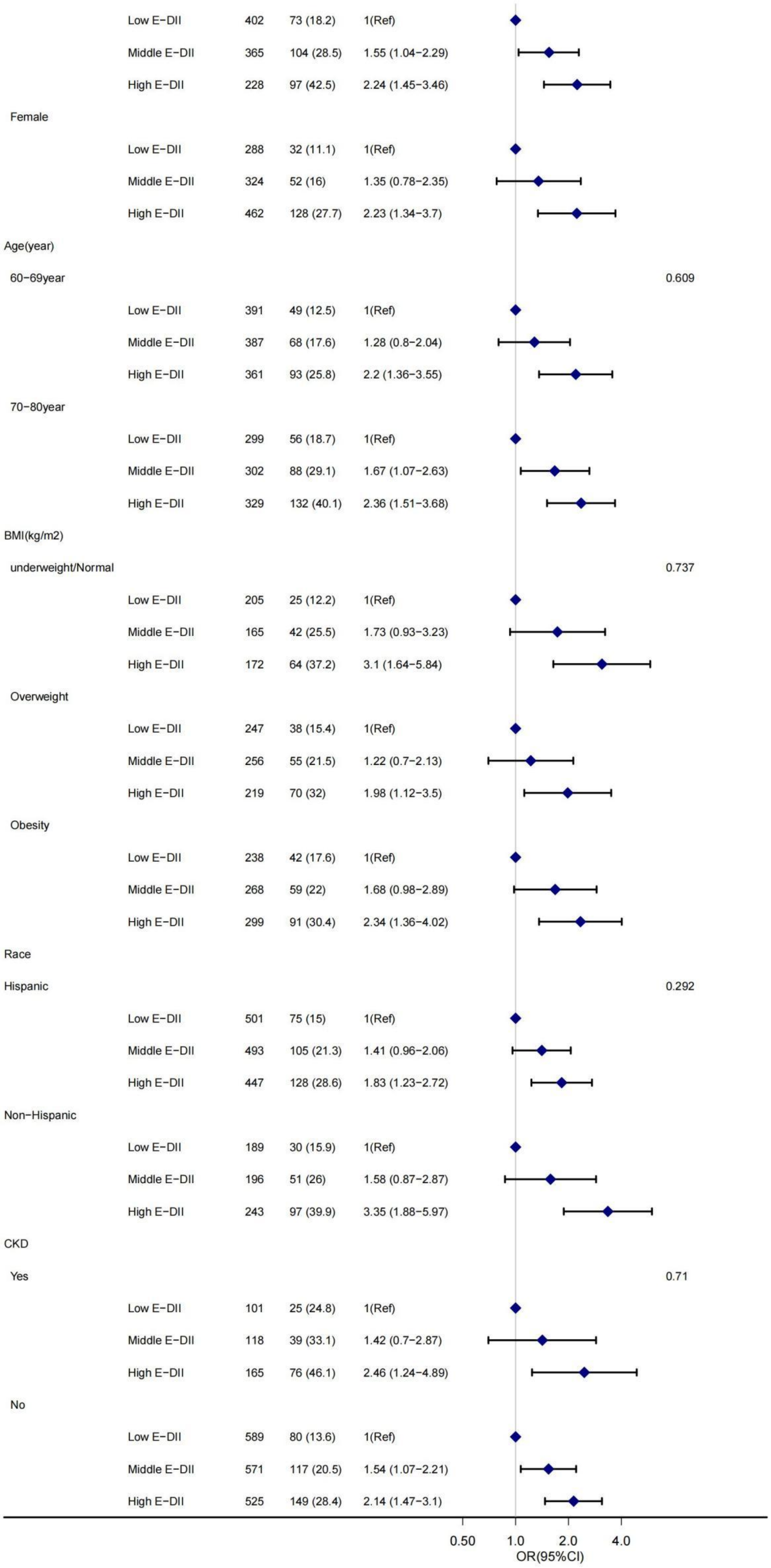

Supplement: Supplementary file 1 [file Data_Sheet_1.PDF]
